# Do methods of hospital pre-alerts influence the on-scene times for acute pre-hospital stroke patients? A retrospective observational study

**DOI:** 10.29045/14784726.2021.9.6.2.19

**Published:** 2021-09-01

**Authors:** Jacob Gunn

**Affiliations:** Great North Air Ambulance Service

**Keywords:** ambulance, hospital, stroke

## Abstract

**Introduction:**

Stroke is one of the leading causes of death and disability worldwide. The ambulance service is often the first medical service to reach an acute stroke patient, and due to the time-critical nature of stroke, a time-critical assessment and rapid transport to a hyper acute stroke unit are essential. As stroke services have been centralised, different hospitals have implemented different pre-alert admission policies that may affect the on-scene time of the attending ambulance crew. The aim of this study is to investigate if the different pre-alert admission policies affect time on scene.

**Method:**

The current study is a retrospective quantitative observational study using data routinely collected by North East Ambulance Service NHS Foundation Trust. The time on scene was divided into two variables; group one was a telephone pre-alert in which a telephone discussion with the receiving hospital is required before they accept admission of the patient. Group two was a radio-style pre-alert in which the attending clinician makes an autonomous decision on the receiving hospital and alerts them via a short radio message of the incoming patient. These times were then compared to identify if there was any difference between them.

**Results:**

Data on 927 patients over a three-month period, from October to December 2019, who had received the full stroke bundle of care, were within the thrombolysis window and recorded as a stroke by the attending clinician, were split into the variable groups and reported on. The mean time on scene for a telephone call pre-alert was 33 minutes and 19 seconds, with a standard deviation of 13 minutes and 8 seconds. The mean on-scene time for a radio pre-alert was 28 minutes and 24 seconds, with a standard deviation of 11 minutes and 51 seconds.

**Conclusion:**

A pre-alert given via radio instead of via telephone is shown to have a mean time saving of 4 minutes and 55 seconds, representing an important decrease in time which could be beneficial to patients.

## Introduction

Strokes are one of the leading causes of death worldwide, causing over five million estimated yearly deaths (Sheppard et al., 2015). They cause life-changing disability and worsening quality of life (Stroke Association, 2018; Strong et al., 2007), and result in high levels of spending from healthcare providers (National Audit Office, 2005). Ischaemic strokes, in which a thrombus restricts blood flow to areas within the brain, are responsible for around 85% of all strokes, while haemorrhagic strokes are responsible for the remaining 15% (NHS, 2019b). Both types of stroke compromise the flow of blood to the brain and are time-critical medical emergencies requiring rapid access to a CT scanner for neurological imaging, to determine which type of stroke the patient is suffering (Hsieh et al., 2016; Wardlaw et al., 2014). If the stroke is identified as ischaemic, then treatment with thrombolysis or thrombectomy can be initiated at a specialist place of care (Price et al., 2020). Thrombolysis treatment requires the administration of a tPA (tissue-type plasminogen activator) within 4.5 hours of the onset of stroke symptoms (Fischer et al., 2017; NICE, 2019). This catalyses plasminogen conversion into plasmin, breaking down the thrombus and restoring blood flow to the affected areas (NICE, 2012). If the patient receives a CT scan that confirms an ischaemic stroke and treatment with alteplase is given within 90 minutes of onset of symptoms, around 25% of patients could recover fully with no long-lasting disability (Price et al., 2019; Wardlaw et al., 2014). The evidence however shows decreasing effectiveness the longer the delay to treatment, with only around 11% of patients recovering fully if treatment is given at the 4.5-hour limit (Fiehler et al., 2016; Hacke et al., 2008). Thrombectomy requires surgical removal of the thrombus and may be effective in restoring blood flow to the brain where thrombolysis alone may not have been affective (Tawil & Muir, 2017). As with thrombolysis, this intervention is more effective the quicker it can be implemented after symptoms begin (Fiehler et al., 2016; Hsieh et al., 2016).

The consensus regarding improved outcomes when treatment is received quickly is backed by the evidence showing that an acute stroke patient loses around 2 million neurons a minute until treatment is started (Saver, 2006). Worldwide, there has been a large amount of investment in innovation to decrease the time it takes for patients to receive neurological imaging and subsequent treatment once they get to hospital (Fischer et al., 2017). Internationally, some areas have brought the CT scanner to the patient in order to begin treatment immediately (Aoun et al., 2015), however this is expensive. Within the UK, current best practice is to transport the stroke patient to a centralised hyper acute stroke unit (HASU) if available, and within the appropriate timeframe from onset (Haran et al., 2016; Price et al., 2019).

Kendall et al. (2015) identified that when the pre-hospital clinicians inform the HASU they are incoming with an acute stroke patient, and then transfer them directly to the CT scanner, this reduces time taken for the patient to receive treatment from when they arrived at the hospital door. Despite this, the study found no improvement in the time it took for the patient from symptom onset to arrival at the hospital. Further studies have agreed that notification of the HASU prior to transportation improves time from hospital arrival to treatment, allowing specialist staff time to prepare for the patient and to begin the implementation of care (Price et al., 2019; Sheppard et al., 2015), and that standardised communication methods could support improved care and outcomes (Price et al., 2019). Despite the consensus for notifying the HASU prior to transportation, and the evidence for standardised communication methods, there appears to be no uniform communication method for pre-alerting the hospital of an incoming acute stroke patient. While these studies have improved care bundles and enabled direct contact with specialist staff, there is still concern within the research that times to treatment need further improvement (Aoun et al., 2015; Kendall et al., 2015; Yang et al., 2017), and that in some areas there is a need to reverse a trend of increased time to treatment (Haworth & McClelland, 2019). Furthermore, there appears to be a current gap in the literature to explore and adequately explain the amount of time which may be lost while a paramedic is on scene with an acute stroke patient, and some studies have included this as weaknesses in their own research, recommending further research and change to mitigate this (Faiz et al., 2013; Kendall et al., 2015; Yang et al., 2017).

The Ambulance Quality Indicators (NHS, 2019a) audit all English ambulance services for stroke care, and have found the North East Ambulance Service NHS Foundation Trust (NEAS) to be performing below average for the time it takes the patient to arrive at the hospital following the original 999 call. In line with this, a service evaluation of on-scene times within NEAS was carried out and pre-hospital on-scene times for acute stroke patients were found to have risen year on year from 2011 to 2018, despite the limited interventions a pre-hospital clinician can implement prior to transport (Haworth & McClelland, 2019; Lally et al., 2020). The findings stated that the average on-scene times increased yearly, with up to 30 minutes as the average time spent on scene in 2018. London Ambulance Service reported similar on-scene times of 31 minutes in an audit conducted in 2017, showing other trusts are experiencing similar extended on-scene times (London Ambulance Service, 2020). Furthermore, the Sentinel Stroke National Audit Programme (SSNAP) reported that patients nationally are arriving by ambulance progressively later year by year, which is concerning (SSNAP, 2019).

## Research aims

Li et al. (2018) discussed barriers that present pre-hospitally and contribute to on-scene delays for acute stroke patients; one of these barriers is communication. The current study aims to identify if the communication through differing pre-alert policies has an impact on the on-scene times for acute stroke patients. These differing pre-alert policies are:

- A radio message pre-alert passed via ambulance control, who then relay this to the hospital (two hospital trusts). This requires no discussion with the receiving hospital as to whether the patient will be accepted, and so may be performed en route to the HASU, bypassing the local non-specialist Emergency Departments.
- A telephone call direct from the on-scene clinician to the receiving stroke ward (three hospital trusts). This requires a detailed telephone discussion with the receiving HASU to ensure they accept the patient prior to bypassing any local non-specialist Emergency Departments. This may be further complicated by mobile phone signal gaps in rural areas and road/equipment noise, which may require the call to be performed prior to transportation.
- A combination of the two depending on the time of day (one hospital trust).

If a significant difference is found, it may be an area for future improvement and streamlining of services. The hypothesis is that a shorter radio-call style pre-alert used instead of a mobile telephone call direct to the unit may help to decrease time on scene.

## Method

### Design

This research study is a retrospective observational study using quantitative data routinely collected from the electronic patient report form (EPRF) in NEAS. The independent variables were the pre-alert systems used which were split into two groups (radio and telephone), and the dependent variable was time spent on scene. The null hypothesis was that the two differing pre-alert policies would have no impact on time on scene for acute stroke patients.

### Setting

This study focused on NEAS who provide pre-hospital care to around 2.5 million people across the North East of England from Teesside to Northumberland (North East Ambulance Service, 2018), and are often the first healthcare providers accessed by acute stroke patients. Within this area are six HASUs with differing requirements for accepting patient admissions from on-scene NEAS clinicians.

### Materials

All of the data for this study were obtained from the logistics team in NEAS. IBM SPSS Statistics version 26 was used to analyse the data.

### Inclusion/exclusion criteria

The resulting cases were reviewed to ensure suitability for the study.

Inclusion criteria:

- those identified as FAST-test positive by the on-scene clinician as reported on the EPRF;
- paramedic attendance at scene within 4.5 hours of symptom onset;
- transported to a HASU.

Exclusion criteria:

- patients under 18 years of age, due to differing care pathways for paediatrics;
- patients outside of the 4.5-hour window, as they would be excluded from thrombolysis treatment and treated using different non-time critical pathways;
- patients who had stroke mimics recorded by the clinician on scene as the ‘impression’ on the EPRF such as infections, blood glucose problems, traumatic injuries and seizures, as although initially such patients may have been marked as having some stroke symptoms by the call-taking process, once assessed by a clinician a different diagnosis was given;
- patients that did not receive the full bundle of care as recommended by National Ambulance Quality Indicators (AQI), as such patients potentially did not have all mimics excluded, and although speed is important it must be in conjunction with the correct care (Siriwardena et al., 2014);
- the HASU that incorporated both admission policies, as it would not have been possible to accurately conclude which admission policy was used in which cases.

The data requested included:

- main impression recorded by the lead clinician;
- age of the patient;
- onset time of symptoms;
- if the patient was eligible for thrombolysis;
- if they had received the full care bundle;
- the receiving hospital they were sent to;
- amount of time in hours, minutes and seconds that the clinicians were on scene for each of these patients.

### Procedure

NEAS provided three months’ worth of quantitative data for patients who had been classified as a potential stroke patient either by the 999/111 call taker or a clinician on scene; this included the time spent on scene and the protocol used by the HASU. The period of three months was used to mitigate short-term individual spikes or lulls in ambulance demand, which may affect times on scene.

The on-scene time was used instead of total callout time, as it avoided geographical issues such as distance to and from the incident, as well as traffic and other transport difficulties, focusing only on the time the clinician was with the patient at scene.

The on-scene time was taken for each patient in these groups and inputted into SPSS, and an independent samples T test was then used to compare these times on scene. This was then reviewed to determine if there were any significant differences between the two groups, and to test the null hypothesis that differing pre-alert policies would have no effect on time on scene for acute stroke patients.

## Results

The data request resulted in 2618 patient cases over the three-month period. Once the inclusion and exclusion criteria had been applied, this left 927 patient cases which were then sorted into groups, depending on which hospital admission protocol was used. Group A consisted of those patient cases that were admitted following a phone call pre-alert and amounted to 490 patients; Group B consisted of those patient cases that were admitted following a radio pre-alert and amounted to 437 patients.

**Figure 1:**
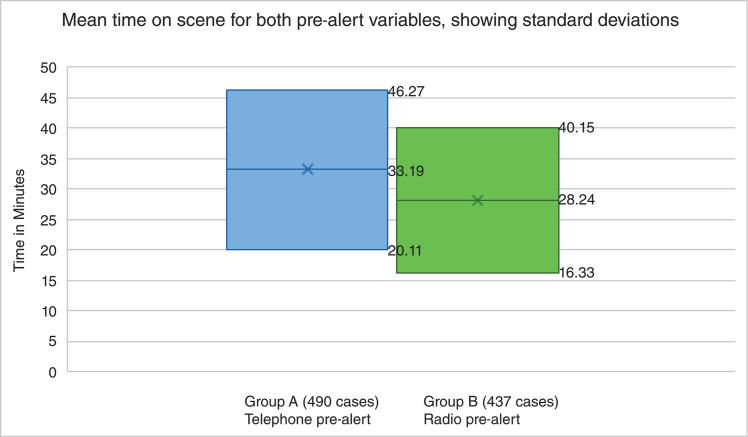
Figure 1. Graphs visually depicting the mean time on scene for both pre-alert variables, with standard deviations included.

The mean time for Group A, the 490 patients taken to a HASU with the required phone call-style pre-alert, was 33 minutes and 19 seconds. This had a standard deviation of 13 minutes and 8 seconds and a standard error mean of 38 seconds. The mean time for Group B, the 437 patients taken to a HASU with a radio pre-alert, was 28 minutes and 24 seconds, with a standard deviation of 11 minutes and 51 seconds and a standard error mean of 34 seconds (see Figure 1).

The difference between the two pre-alert variables was shown to be statistically significant at p < .001. The mean on-scene time differed significantly; t(925) = (5.934), p = < .001. The standard deviations for both groups indicate that the variability of time spent on scene when using the radio pre-alert system is smaller than for the telephone pre-alert system.

## Discussion

### Summary

The results show that within NEAS, when a pre-hospital clinician suspects the patient to have an acute stroke, and gives the correct bundle of care, the amount of time on scene is significantly affected by which pre-notification system is required by the hospital. By using a telephone call pre-alert direct to the ward instead of a radio pre-alert, the average time on scene was increased by 4 minutes and 55 seconds, which could be detrimental to the patient.

### Study significance and relevance

National changes to how ambulances are dispatched, and the centralisation of stroke care, could be expected to increase the amount of time it takes between the initial call for help and the arrival at CT scan (Morris et al., 2014), however there have been no notable changes to how paramedics assess and treat acute stroke patients (Lally et al., 2020; Sheppard et al., 2015). Due to this, the average times on scene should have remained static within NEAS, with the increased travel time accounting for the increasing time delays, however this was not found to be the case. The improvements made in hospital for reducing the times from door to CT and CT to needle are currently able to offset some of this delay (Pulvers & Watson, 2017; SNAAP, 2019), however if improvements are not made in the pre-hospital area then patient outcomes could worsen. SSNAP (2019) acknowledge the importance of this pre-hospital time, stressing the need for improvement and pledging to include more detailed analysis of this in future reports.

The current study demonstrates a considerable difference in on-scene time when using a radio pre-alert policy instead of a telephone pre-alert. Time-on-scene averages are lower than those for the telephone calls system, and more akin to the 2017 NEAS average times on scene of 28 minutes. The differing hospitals served by NEAS have differing preferred departments to which the patient is taken; some review the patient within ED or a stroke ward, and some will meet the ambulance crew and patient at the CT scanner. Direct access to a CT scanner has been found to reduce waiting times for specialist review and treatment, therefore improving outcomes (Hsieh et al., 2016; Price et al., 2019), while the direct-to-ED option will reduce the whole time the patient spends with the ambulance crew but may not have the same improvements for outcomes (Haworth & McClelland, 2019). Delay for the stroke patient whenever it occurs pre-treatment is detrimental. As time on scene can be improved by a radio pre-alert, and patient outcomes can be improved by direct-to-CT or direct-to-ward routes, there is scope for the development of a stroke-specific radio pre-alert that can be used across hospitals, which allows direct access to ward or CT and enables the time saved to be used effectively to help patients.

### Study critique and further research

The current study’s findings provide a compelling argument for the review of pre-alert systems in stroke care, however there are a number of variables which require exploration before reliable conclusions can be drawn. Li et al. (2018) identified five areas in which pre-hospital delays can occur: delays in gaining access to the patient, delays in the assessment and management of the patient, communication delays, extrication and transportation delays and patient refusal (Li et al., 2018). A delay in the on-scene clinician identifying the stroke symptoms may cause some delays, however as found by Harbison et al. (2003), paramedics are as proficient as non-specialist staff in the identification of stroke symptoms. Furthermore, the decision in the current study to include only patient cases receiving the full care bundle was made to ensure a full assessment had been carried out. Further training for pre-hospital staff stressing the importance of effectively managing time on scene once a stroke has been identified could be used in conjunction with a radio pre-alert instead of a telephone call (Puolakka et al., 2016). Individual problems with access to and extraction of patients may also cause delays on scene that would be documented within the notes section of the EPRF. Such factors would require further qualitative exploration in order to understand this variable. Transportation times have inevitably been extended due to the centralisation of stroke care, and are further influenced by variations in time of day, weather, geography and pressure on the service (Elameer et al., 2018; Haworth & McClelland, 2019). Although including the travel time with on-scene time would have represented the total amount of time the paramedic was with the acute stroke patient, it was excluded due to the limited influence that the paramedic can have on these variables. In future, studying a longer timeframe may draw more reliable conclusions that could be used in future research, to develop a more effective admission policy for acute stroke patients.

It may be interesting to conduct interviews with NEAS paramedics to learn of their experiences and views regarding the different pre-alert systems. A narrative is required around the use of different pre-alert systems, to identify their possible benefits and challenges, aside from time spent on scene. For example, it could be identified that some paramedics find clinical value in the longer telephone exchange which they gain within the telephone pre-alert system. It may also be interesting to conduct a study which follows clinical cases all the way through to hospital discharge, to identify if there is any correlation between pre-alert pathway and clinical outcomes for stroke patients.

## Conclusion

Pre-hospital stroke delay is an area in which significant improvements need to be made to complement the improvements made within hospitals for door-to-needle times. The current study shows that in comparison to a radio pre-alert, telephone pre-alert systems may contribute to a significant time delay on scene, which delays the patient receiving time-critical medical care. If a uniform radio pre-alert policy could be implemented across all receiving HASU hospitals accessed by NEAS, alerting them of an incoming acute stroke patient while still allowing for the direct-to-ward and direct-to-CT routes, this could make significant improvements to patient outcomes.

## Author contributions

JG acts as the guarantor for this article.

## Conflict of interest

None declared.

## Ethics

This research was conducted as part of the MSc Advanced Practitioner programme; this was a single-site educational study using anonymised data. Ethical approval for the study was provided by Teesside University in 2020, and the data was accessed through the logistics department of the North East Ambulance Service.

## Funding

None.
